# A Brief Review on Vitamin B_12_ Deficiency Looking at Some Case Study Reports in Adults

**DOI:** 10.3390/ijms22189694

**Published:** 2021-09-07

**Authors:** Elena Azzini, Anna Raguzzini, Angela Polito

**Affiliations:** CREA-Research Centre for Food and Nutrition, Via Ardeatina 546, 00178 Rome, Italy; anna.raguzzini@crea.gov.it

**Keywords:** vitamin B_12_, megaloblastic, neurological disorders, thrombosis

## Abstract

In the era of evidence-based medicine, the randomized clinical trial corresponds to the top step in the qualitative scale of the evidence available in the literature, while small series of cases or the description of individual cases occupy the last place. However, the latter represent an important part of clinical practice and have significantly influenced the evolution of medicine, contributing significantly to the advancement of scientific knowledge. Vitamin B_12_ deficiency shares several common symptoms that affect several tissues and organs with health aliments, so its diagnosis could be unobvious for the broad array of its effects and investigation methods used. In this review, we focused our attention on some case reports related to the vitamin B_12_ deficiency associated to anemia, neurologic disorders, and hyperhomocysteinemia. B_12_ deficiency reversal is simply achieved by prompt therapy, even though it is not the same for several disorders.

## 1. Introduction

During the 1940s, an intensive search for the active factor in liver extracts that prevents pernicious anemia showed that both folate and vitamin B_12_ prevent megaloblastic anemia, but only vitamin B_12_ can prevent neurological complications [1]. It is now known that the two vitamins act jointly in regenerating methionine from homocysteine (Hcy). Hcy accumulates if conversion to methionine is slowed because of a shortage of folate or vitamin B_12_ or both, and a raised plasma Hcy suggests sub-optimal nucleic acid and amino-acid metabolism. It also has direct harmful effects, e.g., it increases the risk of cardiovascular disease through thickening the lining of blood vessels and may also increase the risk of certain cancers and dementia.

As reactions catalyzed by tetrahydrofolate are crucial for cell growth and multi-plication, rapidly dividing cells are particularly vulnerable to a deficiency of either folate or vitamin B_12_. In adults, this affects the bone marrow, causing megaloblastic anemia. In the early embryo, morphogenetic events (particularly those depending on focal rapid cell multiplication) may be affected, increasing the risk of congenital malformation. Single gene disorders caused by rare variants of several enzymes involved in one-carbon transfer cause problems ranging from greatly increased plasma Hcy levels with very early onset cardiovascular disease, to developmental delay and neurological problems, with or without megaloblastic anemia [2]. As previously reported [2], methionine synthase, a vitamin-B_12_-dependent enzyme, catalyzes the folic acid’s donation of a methyl group across methylenetetrahydrofolate reductase (MTHFR). Elevated plasma levels of Hcy can be caused by a deficiency of either vitamin B_12_ or folate, and in human subjects mild (13–24 µM) and moderate (25–60 µM) hyperhomocysteinemia (HHcy) are also associated with mutations of MTHFR genes.

**Figure 1:**
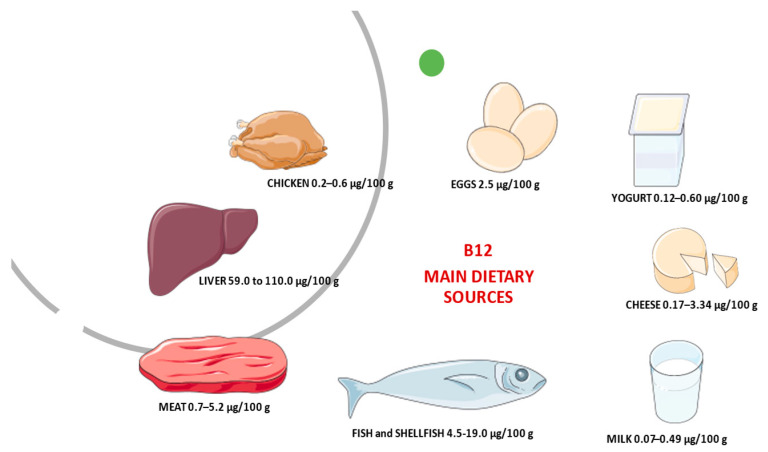
The average content of B_12_ in animal dietary sources.

Vitamin B_12_, also called cobalamin, is synthesized only by micro-organisms [3]. Dietary sources are meat (particularly liver), shellfish, some cheeses, yeast extracts, and the root nodules of legumes (peas, beans, and so on), mainly due to simultaneous bacteria presence in soil and/or their aerial surfaces. Figure 1 reports the main animal dietary B_12_ source and its average content in raw/fresh amounts. Based on the absorption of labeled vitamin B_12_ from some food products, such as chicken meat, rainbow trout, or eggs the bioavailability of vitamin B_12_ is generally assumed to be 40% or 50% for healthy adults without alteration of gastrointestinal functioning [4]. Bioavailability also varies by type of food source. For example, dairy products have a bioavailability of vitamin B_12_ three times higher than meat or fish [5]. Moreover, the bioavailability of vitamin B_12_ from dairy products is considerable [6]. This aspect should take into consideration for vitamin B_12_ recommendations. However, most of the information on B_12_ bioavailability from foods was collected 40–50 years ago, and more recent methods to derive recommendations based on dose–response evidence are still under development [4]. Currently, to maintain a healthy hematological status and serum vitamin B_12_ levels, average daily intakes of vitamin B_12_ from food of 5.94 mcg for men and 3.78 mcg for women aged 20 and older have been recommended. For children aged 2–19 years old, mean daily intakes of vitamin B_12_ from food range from 3.76 mcg to 4.55 mcg [7]. The original estimates of dietary folate and vitamin B_12_ requirements and recommended dietary allowances (RDAs) were based on the amount needed to avoid manifest deficiency disorders (megaloblastic anemia, with sub-acute combined degeneration of the cord in the case of vitamin B_12_ deficiency) and on levels observed in populations. However, these levels do not essentially represent necessary requirements.

As reported by Carmel [8], average total body stores of vitamin B_12_ are 3–5 mg, mostly in the liver. After excretion from bile, it mostly is reabsorbed from the small intestine. It is first bound within the duodenum and jejunum to intrinsic factor produced by gastric parietal cells and is then absorbed in the terminal ileum [9]. Thus, when there is little or no vitamin B_12_ in the diet, stores may last for up to 5–10 years before manifestations of vitamin B_12_ are seen clinically. The etiology of vitamin B_12_ deficiency usually includes causes related to malabsorption, such as autoimmune gastritis (pernicious anemia), celiac disease, inflammatory bowel disease, surgical gastrectomy, gastric bypass, and ileal resection. Less commonly, vitamin B_12_ deficiency can occur due to nutritional habits (strict vegans, breastfed infants born to vegan mothers with decreased dietary intake of animal products), nitrous oxide abuse, Diphyllobothrium latum infection, pancreatic insufficiency, drug interference (metformin, proton pump inhibitors, drug affected purine, and pyrimidine synthesis), inherited disorders affecting intrinsic factor and other inherited disorders including methylmalonic acidemia and transcobalamin II deficiency. Moreover, alcohol abuse could represent a risk for vitamin B_12_ deficiency, for its direct connection with the Hcy concentration, besides reflecting the degree of hepatocytes injury [10,11]. 

The prevalence of vitamin B_12_ deficiency is difficult to ascertain because of diverse etiologies, different evaluation methods (i.e., total serum B_12_ (sB_12_), methylmalonic acid (MMA), holotranscobalamin (holoTC), and total homocysteine (Hcy), as well as different assays (i.e., radioassay or chemiluminescence) [12]. The worldwide prevalence of B_12_ deficiency is estimated to be around 6%, and in Europe it is 1.6–10%. Prevalence is higher in adults over 60 years of age, ranging from 10% to 19% across various countries and is generally higher in women than in men [13]. Pawlak [14] examined the prevalence of vitamin B_12_ deficiency among individuals adhering to vegetarian diets. The reviewed studies show relatively high deficiency prevalence among vegetarians. B_12_ deficiency in infants is about 45%, among the children and adolescents ranging from 0% to 33.3%, and among pregnant women ranging from 17% to 39%, dependent on the trimester. Adults and elderly individuals had a deficiency range of 0–86.5%. Higher deficiency prevalence was reported in vegans than in other vegetarians, while deficiency prevalence of 0% was reported among vegans who consumed vitamin B_12_-fortified foods, highlighting that vitamin B_12_ supplements to ensure adequate vitamin B_12_ intake should be considered in these subjects.

Currently, research suggests that there are disease implications associated with vitamin B_12_ deficiency, especially in vegetarian populations [15,16,17], in pregnancy [18,19], in the elderly [20], and in developing countries [19]. 

In the era of evidence-based medicine (EBM), the randomized clinical trial corresponds to the top step in the qualitative scale of the evidence available in the literature, while small series of cases or the description of individual cases occupy the last place. However, the latter represent an important part of clinical practice and have significantly influenced the evolution of medicine, contributing significantly to the advancement of scientific knowledge. Several reviews describe the results of different clinical trials on the vitamin B_12_ deficiency treatment in different pathological conditions. Here, we focused our attention on some case reports related to the vitamin B_12_ deficiency associated with anemia, neurologic disorders, or HHcy to assess if a common therapeutic approach with vitamin B_12_ supplementation can reverse pathological and/or symptomatologic status. We performed a search in the MEDLINE database (PubMed database; National Library of Medicine, Bethesda, MD) to review the vitamin B_12_ deficiency associated with anaemia, neurologic disorders, or HHcy. All English-language case reports published between 2000 and 2021 in the MEDLINE database were searched using the MeSH search: (cobalamin OR vitamin B_12_) AND (nutrition* OR diet*); Limits Activated: Case Reports, English. The search was further limited to adults and only more representative cases with clear cause/effect evidence will be considered.

## 2. Vitamin B_12_ and Anemia

The role of red blood cells (RBCs) is to transport oxygen through the body. When the number of RBCs is lower than usual body’s tissues and organs do not get enough oxygen, this blood disorder is known as anemia. Among different types of anemia, megaloblastic anemia is characterized by RBCs that are larger than normal and not able to exit the bone marrow to enter the bloodstream and deliver oxygen. However, the molecular basis of these cytomorphological aberrations remains unclear. This clinical condition indicates an altered synthesis of DNA, commonly due to a deficiency of vitamin B_12_ or folic acid, both needed for the production and maturation of RBCs. In particular, the cytoplasm is excessive compared to the nucleus, causing their accumulation in the bone marrow (megaloblastosis) and macrocytosis in the peripheral blood [21]. In fact, the hallmark of megaloblastic anemia is macrocytic anemia (mean corpuscular volume, MCV > 100 fL), often associated with a reduction in the number of mature blood cells (cytopenia). A gold standard for diagnosing megaloblastic anemia is absent, so appropriate clinical and laboratory evaluation allows to establish the correct diagnosis.

Usually, adult patients come to medical attention because of symptoms related to anemia, such as fatigability, lethargy, and exertional breathlessness, pale conjunctiva, paleness and dry lips, and a disturbance of taste. We identify eight case reports all describing vitamin B_12_ deficiency symptoms with etiological causes that appear to be different. A strict vegan 44-year-old woman with a history of anemia on admission presented lethargy, resulting in an inability to continue her job. Without regular medication assumption, except for the contraceptive pill. The biochemical investigations showed levels of haemoglobin (Hb) 110 g/L (reference range 115–160) and MCV 102 fL (range 80–100), and the cause of macrocytic anemia was highlighted by the slightly low levels of vitamin B_12_ 138 pmol/L (reference range 148–600). Test for antibodies to intrinsic factor was negative and oral contraceptive assumption could give a false lower vitamin B_12_ level. Her lethargy and anemia were improved upon three months of treatment with intramuscular hydroxycobalamin 1000 μg three times a week for two weeks. Serum B_12_ increases up to 400 pmol/L, Hb to 133 g/L at three months, as well as an improvement in her lethargy were observed. She was then advised to take oral cyanocobalamin supplements indefinitely as maintenance to prevent further deficiency [22].

Socha et al. [23] reported a case of a 68 years-old man with levels of Hb 6.2 g/dL, MCV 121.5 fL. Bone marrow biopsy presented megaloblastic changes as well as multilineage dysplasia suggestive of myelodysplastic syndrome. Further evaluations confirmed severe vitamin B_12_ deficiency, including a normal serum folate level 18.1 ng/mL (>4.7), a serum vitamin B_12_ level <150 pg/mL (reference range 232–1245); a positive test result for anti-parietal cell antibodies; a normal male karyotype by conventional cytogenetics, a negative hematologic neoplasm next-generation-sequencing panel (62 genes) for disease-associated mutations; in conjunction with normal cytogenetic and next-generation-sequencing panel results, without detectable vitamin B_12_ levels The presence of fatigability, shortness of breath, weight loss, and numbness and tingling of both hands as well as complete blood cell count and neurologic symptoms were improved upon treatment of intramuscular cyanocobalamin (1000 μg) followed by high-dose oral cyanocobalamin (1000 μg/day).

Although glossitis rarely represents another symptom of cobalamin deficiency in addition to those related to anemia, the diagnosis of pernicious anemia with recurrent aphthous stomatitis was the clinical diagnosis of a 71-year-old woman case described by Garcia et al. [24]. Due to the vitamin B_12_ malabsorption, the laboratory investigation showed a MCV of 104.1 fL and low serum vitamin B_12_ (133 pg/mL). Clinical resolution was observed after two months after a treatment of 1.0 mL of hydroxocolabamin intramuscularly twice weekly over four weeks followed by 1.0 mL once weekly for four weeks. Pontes et al. [25] reported an oral manifestation of Vitamin B_12_ deficiency in a 41-year-old strict vegetarian woman for 2.5 years that had not consumed milk, cheese, fish, meat, or eggs during that time. As suggested by the authors, a wide range of oral signs and symptoms may appear in anemic patients because of basic changes in the metabolism of oral epithelial cells. In fact, with regard to the clinical signs of weakness, fatigue, shortness of breath, and neurologic abnormalities, this woman had burning sensation, pale oral mucosa, glossitis with papillary atrophy, and multiple areas of painful erythema on the dorsal surface and lateral borders of the tongue and buccal mucosa were recorded. The hematologic test results showed low levels of Hb (7.2 g/dL), MCV (fL 144), and serum cobalamin (71.8 pmol/L). After 14 days of treatment, comprising a parenteral dose of cobalamin (1000 mg/week hydroxocobalamin administered intramuscularly over 30 days) and 1 mg of folic acid daily for 30 days, the lesions had completely disappeared, as had all other symptoms. A rare presentation of dietary Vitamin B_12_ deficiency anemia was reported by Pahadiya et al. [26]. A 40-year-old male presented a generalized weakness, fatigability, and hyperpigmentation with ecchymosis. His hemogram showed Hb of 9.7 g/dL, MCV 87.8 fL, and normal levels of Vitamin B_12_ equal to 272 pg/mL. Higher levels of lactate dehydrogenase (1250 IU/L) and serum bilirubin 2.43 mg/dL were observed. The authors assumed that these ecchymosed lesions might be due to chronic microhemorrhages in the superficial layer of skin because of thrombocytopenia. The ineffective erythropoiesis and the abnormal red cell membrane are the two pathophysiologic processes featuring in B_12_ deficiency anemia. The resulting reduction in red cell lifespan and the associated hemolysis cause a rising in plasma bilirubin and lactic dehydrogenase (LDH). The cutaneous manifestations of Vitamin B_12_ deficiency including skin hyperpigmentation was reversible after the Vitamin B_12_ supplementation. Treatment with intramuscular hydroxocobalamin 1000 μg daily for five days, subsequently on alternate days for a week, and thereafter weekly for one month restored his hematological parameters to within normal limits and ecchymosis disappeared. 

Surani and Sharma [27] presented a case of pancytopenia caused by an unusual habit of eating pasta. On admission, a 40-year-old female with gradual worsening weakness, intermittent blurry vision, and intermittent paresthesia over three weeks showed the following hematological values: Hb 3.7 g/dL, MCV 105.7 fL, vitamin B_12_ level 16.3 ng/mL (range: 200–900 ng/mL). After transfusion of four units of packed red blood cells, the patient was treated with cyanocobalamin intramuscular injection 1000 mcg daily for one week, then weekly for one month, followed by a monthly injection of 1000 mcg. Four days after admission, the patient improved her Hb to 10.3 g/deciliter (post-transfusion), MCV to 100.2 fL, and after two months, on discharge, Vitamin B_12_ to >2000 ng/mL.

A case of suspected metformin induced cobalamin deficiency (MICD) causing pseudo thrombotic thrombocytopenic purpura was identified as cause of cobalamin deficiency in a 36-year-old female with type 2 diabetes mellitus on metformin for eight years who presented with hemolytic anemia, thrombocytopenia, schistocytes, and mild acute renal failure was studied by Hussain et al. [28]. On admission, laboratory investigations revealed very low serum Vitamin B_12_ levels 91 pg/mL, Hb 3.7 g/dl, and MCV 95.6 fL. As the initial therapy, the authors prescribed intramuscular (IM) cobalamin 1000 μg daily for one week followed by the same dose IM cobalamin weekly for one month. On discharge, Vitamin B_12_ levels increased up to >2000 pg/mL, Hb 8.0 g/dL, MCV 92 fL. Therefore, the authors later prescribed IM cobalamin 1000 μg monthly life long, and at six-month follow-up both Hb 11.9 g/dL and MCV 77 fL improved their values. 

Even if a delay could not impair the diagnostic assurance with neurologic symptoms, their treatment would be started promptly to avoid the risk of irreversibility including extensive sensory defects, gait disturbances, and mental changes. The presence of dizziness and generalized weakness for three days, presence of fever, scleral icterus, conjunctival pallor, and hyperpigmentation of the knuckles of both hands presented in a 48-year-old male case reported by Sasi and Yassin [29]. On admission, the patient’s values were: Hb 6.5 g/dL, MCV 102.6 fL. Hemolysis workup showed indirect hyperbilirubinemia, high lactate dehydrogenase (LDH), low haptoglobin, normal reticulocyte count, and positive direct antiglobulin (DAT). Serum iron studies and thyroid functions were normal, but vitamin B_12_ level was remarkably low <37 pmol/L (reference range 133–675 pmol/L). The intramuscular vitamin B_12_ injection of 1000 μg once weekly improved blood cell counts, which started an upward trend on day 4 after starting the treatment. On discharge, after 10 days, he was asymptomatic with improved levels of Hb 9.8 g/dL, MCV 95.7 fL, and Vitamin B_12_ 369 pmol/L and outpatient appointments for weekly B_12_ injections.

Below, Table 1 summarizes the corresponding laboratory and clinical investigations of the reported cases in this section on anemia and vitamin B_12_ deficiency.

## 3. Vitamin B_12_ and Neurological Disorders

The neurologic manifestations of B_12_ deficiency, including myelopathy, neuropathy, dementia [30,31], and rarely cerebellar ataxia and movement disorders, are difficult to diagnose. In fact, the neurological abnormalities caused by cobalamin deficiency could take place without any hematological or gastrointestinal context [32], and in the absence of anemia or an elevated mean cell volume [33]. The severity of neurological complications may be reversed only by an early treatment after onset, so a timely diagnosis is important. Despite the rapid correction of vitamin B_12_ levels by prompt therapy and early clinical improvement, the recovery of polyneuropathy on nerve conduction could be slow. Deficiency of vitamin B_12_, mostly in vegetarians was found to be associated with depression and adverse neurological function. Berkins [15] points out that the dietary intake of vitamin B_12_ and vitamin B_6_ might have an effect on brain structure. Ralapanawa et al. [34] reported a strict vegetarian 66-year-old female case with demyelinating polyneuropathy without features of anemia (Hb concentration of 12.1 g/dL, RBCs count of 4.39/mm^3^, MCV of 83.3 fL), but very low serum vitamin B_12_ levels (84.90 pg/mL reference range 208–963 pg/mL). To reverse neurological manifestations, after three months of therapy with intramuscular hydroxycobalamine 1000 μg for seven days, weekly for six weeks and thereafter three monthly, the patient showed clinical improvement, with repeated B_12_ levels being elevated up to 308.6 pg/mL. At one- and three-year follow up for nerve conduction study previously absent, early clinical improvement was demonstrated, with a slow recovery of polyneuropathy on nerve conduction studies. Even though vitamin B_12_ deficiency neuropathy is a rare debilitating disease that affects mostly the elderly, young adults with neuropathic symptoms warrant a high index of suspicion. The cause of neurological symptoms resulting from vitamin B_12_ deficiency could be due to the role of methylcobalamin in myelin synthesis. The lack of cobalamin could induce the destruction of myelin sheaths or incorporation of abnormal fatty acids in myelin sheaths, thus leading to impaired neural function and/or transmission. The diagnosis of Vitamin B_12_ deficiency is challenging in resource limited-settings due to limited access to diagnostic tools and unfamiliarity with the disease, owing to its rarity especially in young people. This is the case reported by Ekabe et al. [35]. A 28-year-old sub-Saharan female, presenting peripheral neuropathic symptoms, was treated with oral vitamin B_12_ tablets at doses of 2 mg per day for three months. A diagnosis of vitamin B_12_ deficiency related peripheral neuropathy was made based on her symptoms, ovalo-macrocytosis and hyper-segmented neutrophils on peripheral blood smear. After one month of therapy, an improvement in neurological symptoms was recorded. The authors highlighted the pivotal role of basic investigations like peripheral blood smear for the timely detection and management of vitamin B_12_ associated neurological disease in resource-limited settings. A case of sub-acute combined degeneration (SCD), the most common neurological disorder, in a 33-year-old woman without anemia or macrocytosis leading was diagnosed by Maamar et al. [36] as suspected vitamin B_12_ deficiency, subsequently confirmed by a low serum cobalamin. First investigations revealed Hb 12.1 g/100 mL, MCV 91 fL, other biochemical parameters were within normal limits, while magnetic resonance (MR) imaging of the spine revealed intramedullary hyperintensity in the posterior column of the cervico-dorsal spinal cord, highly suggestive of subacute combined degeneration (SCD). In fact, the patient’s vitamin B_12_ serum level was low (30 pg/mL; reference range 200–700 pg/mL) while serum folate was within the normal range (26 ng/mL; reference range 18–30 ng/mL). Intramuscular administration of B_12_ resulted in correction of the neurological signs (paresthesis and sphincter disorders). At a seven-year follow-up, while still receiving intramuscular vitamin B_12_ monthly, the patient was found to be functionally independent with no neurological deficits. Early spinal MR imaging could support the early diagnosis of SCD of the spinal cord due to Vitamin B_12_ deficiency as reported in the case of a 57-year-old man by Senol et al. [37]. Following clinical and laboratory examinations, the patient was evaluated as cervical myelopathy due to Vitamin B_12_ deficiency (60 pg/mL (reference range 189–883 pg/mL). The symptoms totally disappeared two months after intramuscular supplementation of vitamin B_12_ (1000 µg IM daily for a week and then weekly for six weeks) and the MR imaging abnormalities significantly improved. The same diagnosis of SCD was considered and confirmed by laboratory findings in a 56-year-old man by Srikanth et al. [38]. The patient presented an acute onset of paresthesia involving both hands and feet of 15 days duration, difficulty in walking, and inability to feel the ground for the same period. Neurological examination revealed impairment of sensation of fine touch, pinprick, joint position, and vibration in both hands and feet bilaterally. All the deep tendon reflexes were exaggerated, more so in the lower limbs, with no evidence of motor weakness. Gastric endoscopy and biopsy revealed changes of atrophic gastritis and folic acid and vitamin B_12_ levels in the serum were 7 micrograms and 75 picograms, respectively. Cervical MR image findings were consistent with SCD. MR imaging lesion was completely resolved treating the patient with parenteral administration of vitamin B_12_ and oral folic acid. In summary, SCD is clinically characterized by predominant involvement of the dorsal columns and the lateral columns of the spinal cord, resulting in sensory deficits, paresthesia, weakness, ataxia, and gait disturbance. In some patients, MR imaging shows abnormalities of the spinal cord, indicating demyelination of the posterior column. Early diagnosis and treatment play an important role in the reversibility of neurological deficits. Delayed treatment results in irreversible disabling neurological impairment, such as spasticity and paraplegia.

The seizures rarely occur in patients with vitamin B_12_ deficiency and the molecular mechanisms involving cobalamin in epileptogenesis are unknown. However, Kumar [39] reported this unusual symptom of vitamin B_12_ deficiency in a 26-year-old man. A diagnosis of vitamin B_12_ deficiency with multiple neuropsychiatric manifestations, namely dementia, psychosis, seizures, and myeloneuropathy, was considered. Investigations confirmed the suspicion Hb 13.2 g/dL, and MCV 114 fL. Serum B_12_ assay was 26 pg/mL and folate levels were 28 ng/mL. His symptoms responded to parenteral vitamin B_12_ therapy started on intramuscular vitamin B_12_ injections. At 24 months follow-up, the seizures disappeared and functionality was independent. Regarding this disturbance, the author highlighted the similarities of cobalamin deficiency with multiple sclerosis and supposed that the probable impairment of cerebral neurons was due to destroyed myelin sheaths, which are more susceptible to the excitatory effects of glutamate. Mavromati and Sentissi [40] report a clinical case of delirium due to vitamin B_12_ deficiency in a vegetarian female 62-year-old. Delirium could have multiple causes, so the initial diagnosis presented various difficulties. Details on neurological symptoms are reported. The laboratory and clinical examinations excluded infectious, vascular, neoplastic, metabolic, and endocrine causes. Her serum vitamin B_12_ level was low (91 pmol/L) and folic acid was normal (22.2 mg/L). The patient was treated with vitamin B_12_ supplementation. The vitamin B_12_ level was normalized one week later (330 pmol/L). A psychiatric examination two weeks after the first evaluation revealed an important diminution of cognitive deficiency and a partial remission of the depressive symptoms (MADRS score 22, MMSE 28/30 and DRS-R-98 4; the clock test was normalized). Four weeks after the episode, there was a total remission of the depressive symptoms (MADRS score: 4) and stable mental status. The cause of the vitamin B_12_ deficiency was attributed to the patient’s strict vegetarianism and this finding underlines the importance of conducting a complete laboratory test panel for delirium, including the blood levels of vitamin B_12_. Table 2 summarizes the corresponding laboratory and clinical investigations of the reported cases in this section on neurological disorders and vitamin B_12_ deficiency.

## 4. Vitamin B_12_ Deficiency and Hyperhomocysteinemia

Vitamin B_12_ deficiency can also lead to HHcy and may be associated with osteoporosis, depression, cognitive decline, and some forms of dementia in the elderly. More recently, vitamin B_12_ deficiency has been reported as common among patients with HHcy and thrombosis [41], although the presence of a direct effect of vitamin deficiency rather than mediated by HHcy or other factors is to clarify. In fact, lifestyle-related factors, such as smoking status, BMI, and physical activity, could interfere between HHcy and the thromboembolism relationship [42]. Moreover, the effect of lowering Hcy levels in patients with intermediate (total Hcy 30–100 µmol/L) or severe HHcy (total Hcy > 100 µmol/L) remains unknown [43]. The cases described below report examples of vitamin B_12_ deficiency and HHcy related to different causes. 

A case of cerebral venous thrombosis secondary to HHcy caused by vitamin B_12_ deficiency in a 32-year-old Indo-Aryan man who followed a strict vegetarian diet is reported by Kapur [44]. The preliminary blood examination revealed macrocytic anemia with hemoglobin of 11.4 g/dL and mean corpuscular volume (MCV) of 110 fL. Peripheral blood film showed macrocytes and macro-ovalocytes with hypersegmented neutrophils; low serum cobalamin levels 68 pg/mL (200–600) with normal folate levels and total serum Hcy levels of 36 μmol/L (5.0–13.9) were observed. In addition to other treatments, the patient received parenteral cyanocobalamin 1000 μg once daily for seven days. Gradually, he regained sensorium, his power improved, and he was discharged on orally administered sodium valproate, warfarin, and methylcobalamin. Repeated investigations undertaken at six months after stopping anticoagulants showed normal serum cobalamin 364 pg/mL (200–600) and fasting total Hcy levels 8.4 μmol/L (5.0–13.9). The authors conclude that HHcy is an independent risk factor for cerebral venous thrombosis in patients with cobalamin deficiency, especially those who follow a strict vegetarian diet, and that HHcy can be easily reversed with vitamin supplementation, cobalamin, and folic acid. 

The cases of four Moroccan patients with acute vein thrombosis of different sites are reported by Ammouri [45]. Three men and one woman of different ages (a 34-year-old man, a 60-year-old man, a 58-year-old man, and a 47-year-old woman) were selected. All patients presented low hemoglobin level (from 8.6 g/dL to 9.5 g/dL), low MCV, low cobalamin plasma level (about 60 pg/mL; normal >120 pg/mL), and high levels of plasma Hcy (50 to 200 μmol/L; normal range <15 µmol/L) with normal folate plasma levels. For all, it pernicious anemia and venous thrombosis secondary to HHcy were evident. First, the authors speculated that normal folate levels may have contributed to the delay in the diagnosis of pernicious anemia, leading to severe HHcy and the consequent development of vascular injury. HHcy could lead to venous thrombosis by several pathways.

For example, the toxic effect of Hcy on the vascular endothelium and on the dotting cascade, as well procoagulant properties of Hcy, including the decrease of antithrombin III binding to endothelial heparan sulfate, an increase of affinity between lipoprotein(a) and fibrin, induction of tissue factor activity in endothelial cells, and inhibition of inactivation of factor V by activated protein. In all patients, clinical and biological abnormalities disappeared upon vitamin B_12_ supplementation. The authors concluded that vitamin B_12_ supplements can rapidly correct HHcy avoiding and preventing thrombotic events.

Tanaka et al. [43] reported a case of a 39-year-old man with inferior vena cava (IVC) thrombus. The analysis of risk factors of venous thromboembolism shown HHcy (total Hcy 83.1 µmol/L; normal range 5–15 µmol/L) due to an unbalanced diet with a deficiency of folic acid and vitamin B_12_. The patient was treated with both folic acid and vitamin B_6_/B_12_ supplementation in association with warfarin, inducing a significant resolution of thrombus after four weeks and no evidence of recurrent IVC thrombus at six months. The authors concluded that B vitamins and folic acid therapy might be effective in patients with severe HHcy.

An interesting case of a 43-year-old man presenting with a two-week history of painless ascending sensory disturbances, suspected to be suffering from acute inflammatory polyneuropathy, is reported by Ulrich et al. [46]. On clinical examination, deep tendon reflexes were preserved, muscle strength was 5/5 everywhere, and gait was ataxic. Initial laboratory assessment showed nearly normal holotranscobalamin (43 pmol/L; pmol/L normal >50 pmol/L), suggesting no vitamin B_12_ deficiency. Surprisingly, further investigation showed high Hcy (48.5 µmol/L; normal <10 µmol/L), suggesting an impairment of vitamin B12-dependent metabolism leading to the diagnosis of SCD. The patient remembered having taken tablets containing cobalamin for three days before hospitalization. The authors concluded that holotranscobalamin can be rapidly normalized during supplementation and the analysis of methylmalonic acid and Hcy might help to detect B_12_ deficiency in patients who recently started supplementation.

A case of a 24-year-old male with unprovoked bilateral submassive pulmonary emboli with a high level of Hcy without anemia is reported by Kovalenko et al. [47] Complete blood count showed a MCV of 104fL without anemia, and Hcy level was 41.3 μmol/L (normal 4.0–13.7 μmol/L). Workup for macrocytosis was notable for low vitamin B_12_ (72 pg/mL) and folate (2.1 ng/mL) levels. After vitamin B_12_ supplementation, serum Hcy levels did not decrease to normal values. The authors speculated that a poor absorption of B vitamins due to a small bowel resection two years before and excessive alcohol consumption could have impaired the results. Another case associated with alcoholism was previously described by Goette et al. [48]. The authors described a rare case of a 32-year-old man with severe HHcy underlying a probable cause of thromboembolic complications. The patient did not have a history of cardiovascular disease, but he had at least a six-month history of alcohol abuse at least six months before hospital admission. Laboratory assays showed abnormalities in liver functions, vitamin B_12_ (226 pg/mL; normal range 150–675 pg/mL) and folate (1.6 μg/L; normal range 1.4–11.8 μg/L) were low but within normal range, while serum Hcy was at least 12 times higher than normal (173 μmol/L). The patient was treated with 5 mg oral folic acid and 20 mg oral vitamin B_6_ daily. Vitamin supplementation was then adapted and integrated with other drugs, such as weight-adapted low molecular weight heparin and L-arginine. For some patients, the authors suggested the screening for HHcy in association with endothelial dysfunction markers as appropriate. 

Ruscin et al. [49] illustrated the case of a 78-year-old nonvegetarian white woman with gastroesophageal reflux treated for long-term with histamine(2) (H(2))-receptor antagonists and a proton-pump inhibitor (PPI). During treatment, vitamin B_12_ dropped from normal values (413 pg/mL) to 256 pg/mL; methylmalonic acid (MMA) and Hcy were elevated at 757 nmol/L and 27.3 micromol/L, respectively, serum folate was within the normal range (4.9 ng/mL), and serum creatinine was slightly elevated at 1.4 mg/dL. In addition, no renal dysfunction was present. After oral treatment with vitamin B_12_ (1000 microg/d), MMA and Hcy concentrations decreased dramatically. The authors speculated vitamin B_12_ deficiency because of cobalamin malabsorption from food intake due to drug interference, suggesting vitamin B_12_ status monitoring in patients taking these medications for an extended time, particularly >4 years.

As known, elevated plasma Hcy is involved in cognitive decline, including Alzheimer’s disease, mild cognitive impairment, and dementia, especially in elderly subjects. McCaddon [50] reported seven cases of older patients (four women aged 78 years, 84 years, 77 years and 87 years, 84 years old, and two men 71 and 75 years old). They presented with cognitive impairment and/or depression, dementia, etc. Each had different vitamin B_12_ status with HHcy. Treatment with N-acetylcysteine, together with B vitamin supplements, improves cognitive status in hyperhomocysteinemic patients. The authors concluded that it could be important to evaluate inadequate vitamin B_12_ and folate metabolism in subjects with cognitive diseases, underlining the importance of clinical trials to evaluate the beneficial effects of a synergistic approach to cognitively impaired hyperhomocysteinaemic patients.

Table 3 shows the corresponding laboratory and clinical investigations of the reported cases in this section on HHcy and vitamin B_12_ deficiency.

## 5. Conclusions

Low B_12_ status is a risk factor of megaloblastic anemia, various neuropsychiatric symptoms, and other clinical manifestations. The nutritional guideline recommends the nutrient amounts to be consumed as part of a normal diet to ensure health and safety at each stage of life. In the presence of adequate consumption, any factor influencing their absorption or utilization should be considered. Vitamin B_12_ can be stored in relatively large quantities and its degradation is slow. So, an inadequate intake corresponds to longstanding vegetarians or vegans without any supplement replacement. When the etiology is dietary deficiency, the mainstay of treatment is vitamin B_12_ supplementation. Recently [51], the British Society for Haematology (BSH) issued an update regarding the guidance on Vitamin B_12_ replacement. Where Vitamin B_12_ deficiency is not thought to be diet-related, i.e., due to pernicious anemia, prior gastrectomy, bariatric surgery, achlorhydria, pancreatic insufficiency, short bowel syndrome, bacterial overgrowth, or inflammatory bowel disease, the administration of hydroxocobalamin 1 mg IM every 2–3 months for life is recommended, in addition to special advice during the COVID-19 pandemic for patients established on IM hydroxocobalamin. Where B_12_ deficiency is thought to be diet-related, people should either take oral cyanocobalamin tablets 50–150 micrograms daily between meals or have a twice-yearly hydroxocobalamin 1 mg injection. In vegans, treatment may need to be life-long, whereas in other people with dietary deficiency, replacement treatment can be stopped once the vitamin B_12_ levels have been corrected and the diet has improved. As shown by the case reports above, B_12_ deficiency reverse is simply addressed by prompt therapy, even though it is not the same for several disorders. In the presence of adequate intake, vitamin B_12_ deficiency shares several common symptoms that affect several tissues and organs with health aliments, so its diagnosis could be unobvious for the broad array of its effects and investigation methods used. Screening for vitamin B12 deficiency is generally not recommended in average risk subjects. Case reports emphasize the importance of conducting a wide range of laboratory tests, including an evaluation of vitamin blood levels. Even though case reports may be considered as approaches to personalized therapy based on clinical practice, they could account for important information regarding uncommon events as well as stimulate new hypotheses, and thus may support the emergence of new research. Moreover, under specific conditions, other diagnostic tests should not be neglected. For example, the spinal MR imaging could represent a differential diagnosis of symmetrical posterior spinal cord lesions, some of which are not well known. Because the degree of resolution of the clinical symptoms in B_12_ deficiency depends on early detection, MR findings should not be overlooked. Particularly with respect to neurological damage, several questions remain unanswered concerning B_12_ deficiency, and newer genetic analysis and the effects of the microbiome may represent interesting areas of investigation for evaluating the variability of B_12_ deficiency.

**Figure 2:**
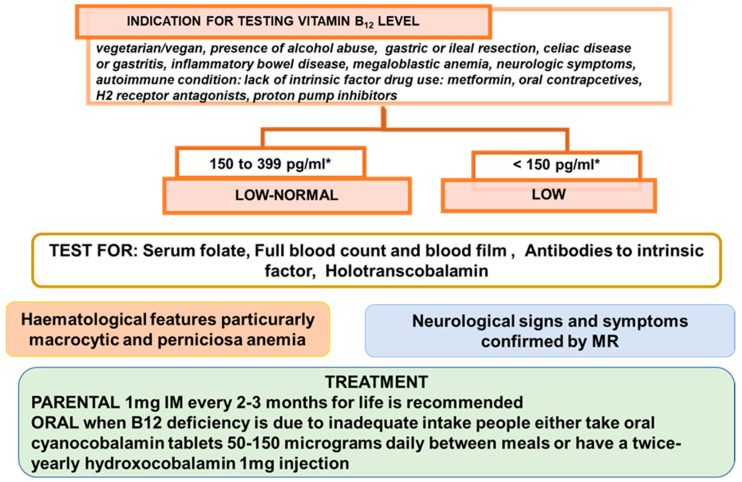
Main conclusions. * According with reference range provided by the local laboratory.

Below, Figure 2 outlines the main conclusion of this brief review.

## Figures and Tables

**Table 1:** Laboratory and clinical investigations of the reported cases in the section on vitamin B_12_ deficiency and anemia, with main changes after treatment.

References	Laboratory and Clinical Investigation	Main Changes after Treatment
Socha et al. [23]	Full blood profile, Folate levels, Vitamin B_12_ levels Parietal cell antibody, Conventional cytogenetics, Hematologic neoplasm next-generation-sequencing panel (62 genes) for disease-associated mutations.	Case 1: Abnormal complete blood cell count findings improved, as did neurologic symptoms. Case 2: rapid improvement of hematologic symptoms and slower but complete resolution of neurologic symptoms.
Garcia et al. [24]	Hb, MCV, Folate levels, iron, ferritin, vitamins B_2_, B_6_, and B_12_ levels, gastroduodenoscopy and gastric biopsy, Antibodies against intrinsic factor and Helicobacter pylori detection	At 12 months the patient was free of the Recurrent aphthous stomatitis with normal levels of hemoglobin, MCV, and vitamin B_12_.
Pontes et al. [25]	Full blood count, Folate levels and vitamin B_12_ levels	After 14 days of treatment complete remission of all symptoms.
Pahadiya et al. [26]	Full blood profile, vitamin B_12_ levels, LDH, bilirubin Bone marrow aspiration, antinuclear antibody and Coomb’s test, Coagulation profile, iron profile Renal function tests, urinalysis and electrolytes Gastroscopy, electrocardiograph, ultrasonography of abdomen, and chest X-ray	At the follow-up of 1 month, hematological parameters were within normal limits and ecchymosis disappeared.
Surani and Sharma [27]	Full blood profile, Folate levels, vitamin B_12_ levels	Hemoglobin improved to 10.3 gm/dL after four days. Complete blood count showed a complete resolution of pancytopenia at two months follow up. Vitamin B_12_ and folate level normalized.
Hussain et al. [28].	Full blood profile, Vitamin B_12_, Folate, Haptoglobin, MMA, Intrinsic factor antibody	At six-month follow-up clinical and laboratory analysis improvement (e.g., hemoglobin improved to 11.9 gm/d).
Sasi and Yassin [29].	Full blood profile, B_12_ level, Bilirubin, LDH Haptoglobin, direct antiglobulin (DAT) Serum iron, thyroid functions	Blood cell counts started showing an upward trend on day 4 after starting the treatment. On discharge, after 10 days of hospital stay, improvement of blood profile and vitamin B_12_ (from values <37 pmol/L to 369 pmol/L), remission of all symptoms.

**Table 2:** Laboratory and clinical investigations of the reported cases in the section on vitamin B_12_ and neurological disorders, with main changes after treatment.

References	Laboratory and Clinical Investigations	Main Changes after Treatment
Ralapanawa et al. [34]	Full blood profile, serum creatinine, plasma glucose, thyroid stimulating hormone levels, vitamin B_12_ levels, nerve conduction studies	After 3 months, clinical improvement, with repeated B_12_ levels being elevated up to 308.6 pg/mL. Follow up at 1 and 3 years showed improvement of nerve conduction.
Ekabe et al. [35]	Full blood profile, HIV test, Treponema pallidum hematoglutinin assay (TPHA), erythrocyte sedimentation rate and peripheral blood smear analysis, stool exam and urinalysis	At 1 months follow up good clinical recovery, improvement in neurological symptoms and a follow up MCV of 97 fl, red blood cell count of 4.1 million/µL, and reticulocyte count of 0.95%.
Maamar et al. [36]	Full blood profile, Somatosensorial evoked potential (SEP), MRI, vitamin B_12_ levels, Folate levels, bone marrow biopsy	Correction of the neurological signs (paresthesis and sphincter disorders).
Senol et al. [37]	Blood glucose, AST, ALT, blood urea nitrogen, creatinine, Hb, MCV, white blood cell count, sedimentation rate, Vitamin B_12_ levels, HbA1C level, Somatosensorial evoked potential (SEP), Electromyography, Gastric endoscopy and biopsy, Brain MR, Cervical spine MR imaging	At two months follow up complete resolution of symptoms, MR imaging abnormalities significantly improved; impairment of the Somatosensorial evoked potential continued.
Srikanth et al. [38]	Full blood profile, bone marrow biopsy, Visual evoked potential and brain stem evoked potential studies, Gastric endoscopy and biopsy, workups for infections, para infectious myelitis, multiple sclerosis and connective tissue disorders, Folate levels, vitamin B_12_ levels, cervical MR examination	At 10 months follow-up, MRI revealed total resolution of cord abnormality.
Kumar [39]	Full blood profile, bone marrow biopsy, vitamin B_12_ levels, Folate levels, Anti-intrinsic factor antibody Gastric endoscopy and biopsy, Brain CT scan, EEG	At 24 months follow-up resolution of seizure and functional independence.
Mavromati & Sentissi [40]	Full blood profile, Electrolytes, vitamin B_12_ levels, Folate levels, Lyme’s test brain MRI, Neuropsychiatric tests	At 1 week normalization of vitamin B_12_ level (330 pmol/L); at 2 weeks important diminution of the cognitive deficiency and a partial remission of the depressive symptoms (MADRS score 22, MMSE 28/30 and DRS-R-98 4; the clock test was normalised). Four weeks after the episode total remission of the depressive symptoms (MADRS score: 4) and stable mental status.

**Table 3:** Laboratory and clinical investigations of the reported cases in the section on vitamin B_12_ deficiency and HHcy, with main changes after treatment.

References	Laboratory and Clinical Investigation	Main Changes after Treatment
Tanaka et al. [43]	Full blood profile, prothrombin time, protein C, protein S levels, total homocysteine, folic acid, vitamin B_12_ (Antinuclear antibody (fluorescent antibody technique), immunoglobulin G anticardiolipin antibodies (IgG ACA), phospholipid (GPL), Lupus anticoagulant (diluted Russell’s viper venom time rate). Tumor marker, carcinoembryonic antigen (CEA carbohydrate antigen 19-9, and a-fetoprotein (AFP), CT	Serum homocysteine level decreased (total homocysteine: 12.4 mmol/L), and swelling of his leg improved with significant resolution of thrombus by CT.
Kapur [44]	Full blood profile, Peripheral blood film, serum cobalamin levels, prothrombin time, protein S, antithrombin III, fibrinogen levels, factor V Leiden assay and prothrombin gene mutation, fasting total serum homocysteine levels, neurological examination, Cerebrospinal fluid examination, CT, MRI	Significant improvement of neurological symptoms. At 6 months normal serum cobalamin 364 pg/mL (200–600) and fasting total homocysteine levels 8.4 μmol/L (5.0–13.9). The rest of the thrombophilia profile was within normal limits.
Ammouri [45]	Full blood profile, prothrombin time, partial thromboplastin time, fibrinogen level, protein C, protein S levels, antithrombin III function, genetic testing for factor V Leiden and factor II mutation, plasma homocysteine level, cobalamin plasma level, folate plasma, antibodies to intrinsic factor, bone marrow biopsy, chest radiographs, ECG, TC, Ultrasonography	Case 1: After a 1-year follow up total remission of psychiatric disorders and thrombotic events. Hemoglobin and homocysteine plasma levels were within normal range. Case 2: At 6-month follow-up period, hemoglobin and homocysteine plasma levels were within normal range. No thrombotic events for 3 years after the follow-up. Case 3: At 6-month follow-up period, hemoglobin and homocysteine plasma levels were within normal range. No thrombotic events during 4 years of follow-up. Case 4: At 3-year follow-up no psychiatric disorders and thrombotic events. Homocysteine plasma level was within normal range.
Ulrich [46].	Full blood profile, holotranscobalamin plasma levels, total homocysteine, MMA, Folate, zinc and copper, Electroneurography, CT, MRI.	Cyanocobalamin, MMA and homocysteine levels continuously decreased, and were normal again after 1 month; improvement of sensory disturbances and gait ataxia; At 2 months follow-up MRI showed significant regression of the dorsal column hyperintensities.
Kovalenko et al. [47]	Full blood profile, troponin, blood urea nitrogen, creatinine, serum electrolytes, B-type natriuretic peptide level, Factor V Leiden, prothrombin mutation, cardiolipin antibody, lupus anticoagulant, anti-B_2_ glycoprotein, protein C, protein S levels, Homocysteine level, vitamin B_12_, folate levels, chest radiographs, ECG, echocardiogram, Pulmonary angiography	Serum Hcy levels did not decrease to normal values.
Goette et al. [48].	Full blood profile, lipid profile, Liver function tests (γ-glutamyl transpeptidase, Alanine transaminase and aspartate aminotransferase, bilirubin), activated partial thromboplastin time, international normalized ratio, thrombin time, activated recalcification, fibrinogen, clotting factors II, XII and VIII levels, protein C, protein S, anti-phospholipid antibodies, vitamin B_12_, folate, Hcy, analyses of cofactors and enzymes involved in homocysteine metabolism, serum levels of 8-isoprostaglandin F2α dimethy larginine (ADMA), Plasma concentrations of arginine and symmetric dimethyl arginine (SDMA), serum level of creatinine, urine analysis 5,10-methylenetetrahydrofolate reductase (MTHFR) gene, TC, computed tomography angiography, ultrasound, echocardiogram	At 2 weeks follow-up level of homocysteine had decreased to 57.6 μmol/L. Three weeks later homocysteine level was 18.1 μmol/L, and after 3 months it was 5.5 μmol/L. After completing his the following metabolites had decreased: ADMA, to 0.363 μmol/L; SDMA, to 0.32 μmol/L; arginine, to 62.8 μmol/L; light reflex rheography and oscillography shown normal perfusion; improvement of pain, paraesthesia in right leg and increasing of pain-free walking distance.
Ruscin et al. [49]	Full blood profile, vitamin B_12_, methylmalonic acid (MMA), total serum homocysteine, serum folate, serum creatinine, renal function test.	At first follow-up vitamin B_12_ has increased, MMA and HCYS was reduced at 351 nmol/L and 23.7 µmol/L respectively. At second follow-up vitamin B_12_ was normal; MMA and HCYS were further reduced but remain slight elevated.
McCaddon [50]	Full blood profile, vitamin B_12_, serum and red cell folate, plasma folate, parietal cell antibodies, total serum homocysteine, cognitive tests.	Case 1: improvement in memory and cognitive tests. Case 2: Within one month tHcy fell to 7.5 μmol/L; no significant cognitive deficits. Case 3: No improvement; the patient died from a bronchopneumonia several weeks later. Case 4: At six-months follow up tHcy fell to 6.6 μmol/L; marked improvement in general behaviour observed also three years later. Case 5: improvement in cognitive tests. Case 6: tHcy fell to 9.6 μmol/L; improvement in cognitive tests. Case 7: At one month follow-up tHcy fell to 8.3 μmol/L; improvement in cognitive tests. At one year follow up MRI scan showed no significant progression in the extent or size of the focal areas of abnormality in the deep white matter, and no change in ventricular configuration.

## Abbreviations

ADMA	8-isoprostaglandin F2α dimethy larginine
CT	computed tomography
DRS-R-98	Delirium Rating Scale—Revised-98
Hb	hemoglobin
Hcy	homocysteine
HHcy	hyperhomocysteinemia
MICD	metformin induced cobalamin deficiency
MADRS	Montgomery Asberg Depression rating scale
MCV	mean corpuscular volume
MMA	methylmalonic acid
MR	magnetic resonance
MMSE	Mini-Mental State Examination
MTHFR	5,10-methylenetetrahydrofolate reductase gene
PPI	proton-pump inhibitor
RDAs	recommended dietary allowances
RBCs	red blood cells
SCD	sub-acute combined degeneration
SDMA	arginine and symmetric dimethyl arginine
TPHA	Treponema pallidum hematoglutinin assay
